# Traditional Japanese medicine Kamikihito ameliorates sucrose preference, chronic inflammation and obesity induced by a high fat diet in middle-aged mice

**DOI:** 10.3389/fendo.2024.1387964

**Published:** 2024-04-29

**Authors:** Yuko Maejima, Shoko Yokota, Megumi Yamachi, Shingen Misaka, Tomoyuki Ono, Hiroaki Oizumi, Keita Mizuno, Shizu Hidema, Katsuhiko Nishimori, Masato Aoyama, Heidi de Wet, Kenju Shimomura

**Affiliations:** ^1^ Department of Bioregulation and Pharmacological Medicine, Fukushima Medical University School of Medicine, Fukushima, Japan; ^2^ Department of Obesity and Inflammation research, Fukushima Medical University School of Medicine, Fukushima, Japan; ^3^ Tsumura Kampo Research Laboratories, Kampo Research and Development Division, Tsumura & Co., Ibaraki, Japan; ^4^ Department of Animal Science, Faculty of Agriculture, Utsunomiya University, Utsunomiya, Japan; ^5^ Department of Physiology, Anatomy and Genetics, University of Oxford, Oxford, United Kingdom

**Keywords:** high fat diet, inflammatory cytokine, Kamikihito, sucrose preference, traditional Japanese medicine, oxytocin receptor, oxytocin

## Abstract

The high prevalence of obesity has become a pressing global public health problem and there exists a strong association between increased BMI and mortality at a BMI of 25 kg/m^2^ or higher. The prevalence of obesity is higher among middle-aged adults than among younger groups and the combination of aging and obesity exacerbate systemic inflammation. Increased inflammatory cytokines such as interleukin 6 and tumor necrosis factor alpha (TNFα) are hallmarks of obesity, and promote the secretion of hepatic C-reactive protein (CRP) which further induces systematic inflammation. The neuropeptide oxytocin has been shown to have anti-obesity and anti-inflammation effects, and also suppress sweet-tasting carbohydrate consumption in mammals. Previously, we have shown that the Japanese herbal medicine Kamikihito (KKT), which is used to treat neuropsychological stress disorders in Japan, functions as an oxytocin receptors agonist. In the present study, we further investigated the effect of KKT on body weight (BW), food intake, inflammation, and sweet preferences in middle-aged obese mice. KKT oral administration for 12 days decreased the expression of pro-inflammatory cytokines in the liver, and the plasma CRP and TNFα levels in obese mice. The effect of KKT administration was found to be different between male and female mice. In the absence of sucrose, KKT administration decreased food intake only in male mice. However, while having access to a 30% sucrose solution, both BW and food intake was decreased by KKT administration in male and female mice; but sucrose intake was decreased in female mice alone. In addition, KKT administration decreased sucrose intake in oxytocin deficient lean mice, but not in the WT lean mice. The present study demonstrates that KKT ameliorates chronic inflammation, which is strongly associated with aging and obesity, and decreases food intake in male mice as well as sucrose intake in female mice; in an oxytocin receptor dependent manner.

## 1 Introduction

The high prevalence of overweight and obesity is a global problem (1) and there exists a strong association between increased BMI (over 25 kg/m^2^) and mortality (2). The prevalence of obesity is higher among middle-aged adults (40-59 yrs) than among younger groups (20-39 yrs) (3). It is known that obesity with an accumulation of visceral fat mass induces chronic inflammation in the brain, adipose tissue, and the liver, ultimately leading to development of further obesity, steatohepatitis, and arteriosclerosis (4). Clinically, high-sensitive C-reactive protein (hs-CRP) is known to be a marker of inflammation and is predictive for myocardial infarction, stroke, and the aggravation of metabolic syndrome (5). C-reactive protein (CRP) is synthesized and secreted in hepatocytes and its transcription is mainly under IL-6 control (6). Increased plasma concentrations of CRP is also associated with aging (7). It has also been reported that both obese and elderly individuals have higher levels of circulating inflammatory markers (8). The combination of obesity and aging is additive leading to chronic, low-grade systemic inflammation (9). Accumulated abdominal adipose tissue secretes pro-inflammatory cytokines, such as IL-1β, TNFα and IL-6 and induces low-grade inflammation (10). Furthermore, this obesity induced pro-inflammatory cytokines lead to liver inflammation, which eventually culminates in non-alcoholic fatty liver disease (NAFLD) (10). NAFLD may lead to further complications such as hepatic steatosis, non-alcoholic steatohepatitis (NASH), fibrosis, cirrhosis, liver failure, and hepatocarcinoma (11).

Oxytocin is a neuropeptide composed of nine amino acids, derived from hypothalamic paraventricular nucleus and supraoptic nucleus. Originally discovered as the hormone that promotes parturition and milk ejection during lactation in females (12), recent work demonstrates a number of new physiological functions for this hormone peptide, such as increasing trust and bonding in humans (13), a decrease of stress and anxiety (14), and an anti-inflammatory effect on microglia (15).

Oxytocin and OXTR mediated signaling also has strong anti-obesity effects. Both OXTR- and oxytocin-deficient mice developed obesity, without changes in food intake and locomotor activity (16, 17), suggesting that the physiological role of oxytocin is to increase energy expenditure (18). On the other hand, oxytocin treatment has been shown to decrease food intake, body weight (BW), abdominal and subcutaneous fat mass, and improve insulin secretion in obese mice (19). The anti-obesity effects were further confirmed in both monkeys and humans (20, 21). Interestingly, oxytocin has been shown to preferentially suppresses the intake of sweet-tasting carbohydrates in mammals (22). This was further confirmed for sucrose intake in rodents and for fructose-sweetened beverage intake in monkeys (20, 23). It has been known that plasma oxytocin levels were decreased by aging (24), as well as obesity (25). Oxytocin plays an important role in muscle regeneration (24) and hepatic regeneration (26) in aged animals.

Obesity is associated with sarcopenia in aged adults, and weight loss induces further muscle mass loss (27). However, nasal oxytocin administration for an 8 week period significantly increased whole lean mass with a trend toward decreasing fat mass in older adults with sarcopenic obesity (27). Therefore, reduction of oxytocin levels may have a significant impact on aging related symptoms, and treatment by oxytocin has potential to improve these metabolic symptoms.

Kamikihito (KKT), a traditional herbal medicine composed of 14 crude drugs, is clinically used in Japan for the treatment of neuropsychological stress disorders including neurosis, amnesia, and insomnia. KKT has been reported to attenuate depressive-like behavior (28), and increase oxytocin secretion in cerebrospinal fluid in rats with acute stress (29). Our previous study clearly demonstrated that KKT activates oxytocin receptors (OXTR) and functions as an agonist (30). It is considered that KKT promotes oxytocin secretion by activating OXTR expressed in oxytocin neurons (30), and may alleviate neuropsychological stress disorders.

Thus, in the present study we examined whether KKT, as an oxytocin agonist, has effects on 1) BW and food intake in a diet-induced obese mouse model, 2) inflammation in adipose and liver tissue in obese mice, 3) sweet preferences in obese mice, and 4) sweet preference in lean oxytocin deficient (*Oxt* ^-/-^) mice.

It is well established that the combination of obesity and aging accelerate systemic inflammation. Therefore, in this study, we specifically focused on these two factors, “age” and “obesity”, which are also prevalent in the typical patient cohort taking KKT supplements. In order to address both “age” and “obesity”, middle-aged diet-induced obese mice were used in this study. We avoided to use of geriatric mice (generally considered as aged over 96 weeks) due to additional, unrelated complications associated with advanced age. However, middle-aged mature mice (10-15 month) is a relevant model as age related body changes are in progress (31). According to previous work, 50-week-old mice would be the equivalent to 38-year-old humans, generally considered the time when aging related change starts (32). Also, the diet-induced obese mice used in this study corresponds to 46.7 years old in male mice, and 28.4-32.2 years old in female mice, respectively.

The vast majority of research published in the field of metabolism involve mice of a much younger age, typically between 12-25 weeks, and it is well known that the protein expression profiles vary drastically between the different stages of life (33). It is therefore essential that mechanistic studies on the systemic effects of drugs should be undertaken in murine models which recapitulate the typical physical state of patients taking the drug, and the contribution of age and body weight should be taken into account. In this study we report for the first time the effect of KKT administration on body weight, inflammation and sucrose preferences in diet-induced obese middle-aged mice.

## 2 Materials and methods

### 2.1 Animals

Six-week-old male and female C57BL/6J mice were purchased from Japan SLC (Shizuoka, Japan). A high fat diet ([HFD]; HFD32; Clea, Osaka, Japan) was given for 54 weeks for male and 31 weeks for female mice. The HFD feeding protocol was adopted to induce severe obesity and to achieve an equivalent body weight between male and female mice. Initial BW were 55.8 ± 1.6 g and 56.2 ± 0.8 g in male and female mice, respectively with no significant differences (T = 0.21, df = 15, *p* > 0.05).

Oxytocin-deficient (*Oxt* ^-/-^), female mice (35–50-week-old) generated by Nishimori et al., were used (34). Mice were fed a standard chow diet [SD] (CE2; Clea, Osaka, Japan). The percentages of kcalories from each ingredient were as follows: SD – protein 20.5%, fat 10.1%; and HFD – protein 20.1%, fat 56.7%.

The animals were kept on a 12-h light/dark cycle (7:00-19:00) with *ad libitum* access to water in individual cages. All experimental procedures and care of animals were carried out according to relevant guidelines and regulations, and were approved by the ethical committee of Fukushima Medical University for Genetic Experiments (Approval number: 306) and Animal Care and Use (Approval number: 2023004).

### 2.2 Protocol for KKT administration in HFD-fed mice

Seven days prior to the start of the experiment, animals were moved to individual cages and habituated for oral gavage. For the wild type (WT) obese mice experimental group, the diet was changed from HFD to SD after starting the experiment. The KKT (500 mg/kg/5ml) was dissolved in distilled water for oral administration. The dose of KKT was based on the prior studies of KKT (200–2000 mg/kg oral administration in mice or rats) (30, 35, 36). KKT (Lot No. 341006900) was kindly provided by Tsumura & Co (Tokyo, Japan). KKT is composed of 14 herbal components (**Supplementary Table 1**); Astragalus Root (3 g, *Astragalus mongholicus Bunge*), Bupleurum Root (3 g, *Bupleurum falcatum L. or Bupleurum scorzonerifolium Willd.*), Jujube Seed (3 g, *Ziziphus jujuba Mill.*), Atractylodes Lancea Rhizome (3 g, *Atractylodes lancea (Thunb.) DC.*), Ginseng (3 g, *Panax ginseng C.A.Mey.*), Poria Sclerotium (3 g, *Erythrococca ulugurensis Radcl.-Sm.*), Longan Aril (3 g, *Dimocarpus longan Lour. or Dimocarpus longan subsp. longan*), Polygala Root (2 g, *Polygala tenuifolia Willd.*), Gardenia Fruit (2 g, *Gardenia jasminoides J.Ellis*), Jujube (2 g, *Ziziphus jujuba Mill.*), Japanese Angelica Root (2 g, *Angelica acutiloba (Siebold & Zucc.) Kitag*. *or Angelica acutiloba* var. *acutiloba*), Glycyrrhiza (1 g, *Glycyrrhiza uralensis Fisch. ex DC. or Glycyrrhiza glabra L.*), Ginger (1 g, *Panax ginseng C.A.Mey.*), and Saussurea Root (1 g, *Dolomiaea costus (Falc.) Kasana & A.K.Pandey*). The plant names have been checked with “World Flora Online” (www.worldfloraonline.org) or MPNS (http://mpns.kew.org). Plant materials were authenticated by identification of external morphology and marker compounds (saikosaponin b2, geniposide, and glycyrrhizinic acid) for plant specimens according to the methods of the Japanese Pharmacopeia and company standards. This drug was prepared as a spray dried powder from a hot water extract (yield 15.6%). KKT was manufactured under strict scientific and quality control, and approved for ethical clinical use by the Ministry of Health, Labor, and Welfare of Japan.

**Figure 1:**
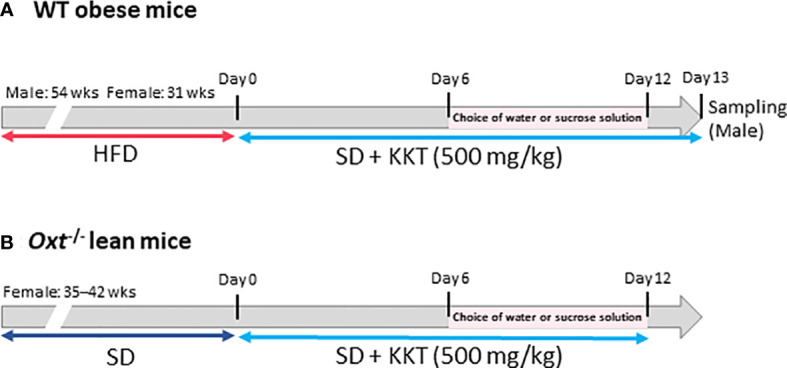
The scheme of this study. **(A)** Six-week-old male and female C57BL/6J mice were fed a high fat diet (HFD); 54 weeks for the male mice and 31 weeks for the female mice. Wild type (WT) obese mice, food was changed from HFD to standard (SD) chow after starting experiment. Kamikihito (KKT: 500 mg/kg/5ml) for oral administration was dissolved in distilled water. Body weight (BW) and food intake were measured for six days under KKT administration. Seven days after starting the experiment, water intake and sucrose water (30% sucrose) were measured, as well as food intake and BW. **(B)** *Oxt***^-/-^** female mice were fed SD chow for 35–42 weeks. KKT (500 mg/kg/5ml) for oral administration was dissolved in distilled water. BW and food intake were measured for six days under KKT administration. Seven days after starting the experiment, water intake and sucrose water (30% sucrose) were measured, as well as food intake and BW.

BW and food intake were measured for six days under KKT administration. Seven days after starting the experiment, water intake and sucrose water (30% sucrose) intake were measured, as well as food intake and BW. The protocol for the WT obese mice is shown in **Figure 1A**, and that for the *Oxt* ^-/-^mice is shown in **Figure 1B**.

### 2.3 Quantitative real−time PCR

Thirteen days after starting the experiment, the WT obese male mice were anesthetized (10 ml/kg, intraperitoneal injection of a mixture of three anesthetic agents [composition; Medetomidine (0.003%, Domitor, Nippon Zenyaku Kogyo Co., Ltd., Koriyama, Japan), midazolam (0.04%, Dormicum, Astellas Pharma Inc., Tokyo, Japan), and butorphanol tartrate [(0.05%, Vetorphale, Meiji Seika Pharma Co., Ltd., Tokyo, Japan)] and blood, liver and mesenteric fat collected.

WT and *Oxt* ^-/-^ female mice (50 weeks) were anesthetized and brains were harvested. Brain tissues were sectioned (0.14 mm to -2.06 mm from bregma) and the hippocampus, hypothalamus and cerebral cortex containing amygdala were further dissected from sections.

Total RNA was isolated from the collected liver and mesenteric fat and each brain region using the RNeasy Mini Kit (QIAGEN, Hilden, Germany) and Monarch RNA Purification Columns (New England BioLabs Inc., MA). C-DNA synthesis was performed using an M-MLV (Thermo Fisher Scientific, MA), RNaseOUT Recombinant Ribonuclease Inhibitor (Thermo Fisher Scientific, MA), and dNTP (Agilent Technologies, TX). A quantitative RT-PCR assay was performed using the TB Green Premix Ex Taq II (Tli RNaseH Plus, Takara Bio Inc., Shiga, Japan). The cycling protocol was: initial denaturation at 95°C for 30 sec, then 35 cycles of 95°C for 5 sec, 56°C for 10 sec, then 72°C for 15 sec. Product accumulation was measured in real time and the mean cycle thresholds were determined. Expression levels of pro-inflammatory cytokines, anti-inflammatory cytokines and OXTR mRNA were calculated using the 2^ΔΔ^CT method of relative quantification and normalized to the housekeeping gene GAPDH. The PCR primers were as follows: IL1β (NM_008361): Fw (GAAGATGGAAAAACGGTTTG), Rev (GTACCAGTTGGGGAACTCTGC), TNFα (NM_001278601): Fw(GCCTCTTCTCATTCCTGCTTG), Rev(CTGATGAGAGGGAGGCCATT), IL-6(NM_031168): Fw(AGACAAAGCCAGAGTCCTTCA), Rev(GGTCCTTAGCCACTCCTTCTG), TGFβ (NM_009369): Fw(GAGCTGCTTATCCCAGATTCA), Rev(GGCAGTGGAGACGTCAGATT), IL-10 (NM_010548): Fw(CAGAGCCACATGCTCCTAGA), Rev(GTCCAGCTGGTCCTTTGTTT), OXTR (NM_001081147): Fw(GCACGGGTCAGTAGTGTCAA), Rev(AAGCTTCTTTGGGCGCATTG), GAPDH (NM_001289726): Fw(TCCACTCACGGCAAATTCAACG), Rev(TAGACTCCACGACATACTCAGC).

### 2.4 The measurement of plasma TNFα and CRP

Blood samples were collected in EDTA tubes and centrifuged at 3000 rpm for 10 min at 4°C. Plasma was collected and TNFα concentrations were measured using a mouse TNFα ELISA kit (430907, Biolegend, CA). Intra-assay and inter-assay variations were 3.8–4.2% and 1.7–3.0%, respectively. Plasma CRP concentrations were measured by mouse high-sensitive (hs)-CRP ELISA kit (KT-095, Kamiya Biomedical Company, WA). Intra-assay and inter-assay variations were under 10%.

### 2.5 Statistical analysis

All data are presented as mean ± SEM. Student’s t-test was used for two-group comparisons. Comparisons of the two groups at each sampling point were analyzed by repeated measures two-way (sampling day×treatment) ANOVA followed by Tukey’s multiple range test. All statistical tests were two-tailed, with P values of 0.05 considered statistically significant.

## 3 Results

### 3.1 KKT administration decreases food intake in obese male, but sucrose intake in obese female mice

**Figure 2:**
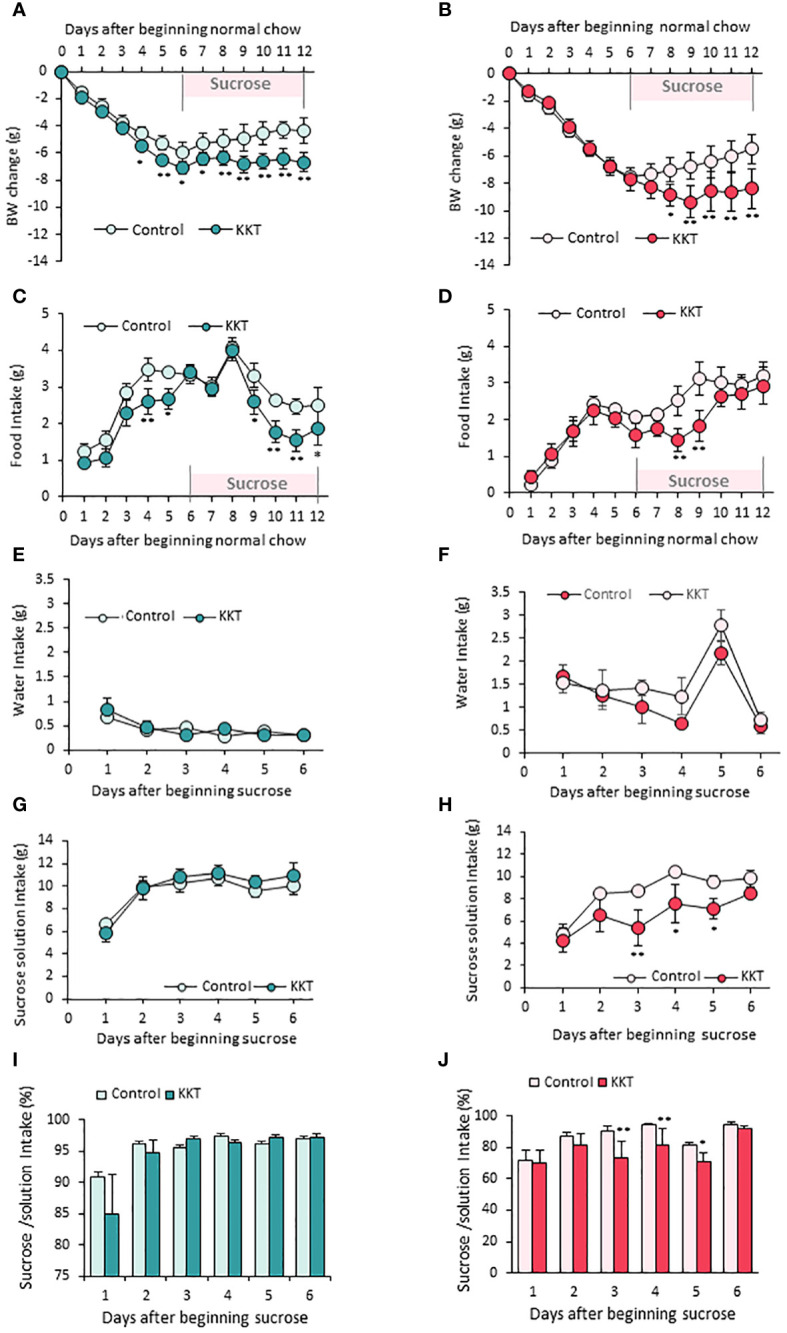
KKT suppresses food intake in male, sucrose intake in female mice. **(A–D)** Body weight change **(A, B)** and food intake **(C, D)** during oral KKT administration in obese male **(A, C)** and obese female **(B, D)** mice. *P < 0.05, **P < 0.01, Repeated measures two-way ANOVA followed by Tukey’s multiple range test. **(E–H)** Water intake **(E, F)** and sucrose solution intake **(G, H)** during sucrose giving term in obese male **(E, G)** and obese female **(F, H)** mice. * P < 0.05, **P < 0.01, Repeated measures two-way ANOVA. followed by Tukey’s multiple range test. **(I, J)** The ratio of sucrose intake ([sucrose intake]/[water + sucrose intake] × 100) in obese male **(I)** and obese female **(J)** mice. *P < 0.05, **P < 0.01, Repeated measures two-way ANOVA followed by Tukey’s multiple range test. **(A, C, E, G, I)** *n* = 5, 6. **(B, D, F, H, J)** *n* = 4, 4.

In order to identify the effect of KKT on standard chow feeding on obese mice, food intake and BW were measured for the first six days under standard chow in HFD induced obese mice as shown in the schematic (**Figure 1A**). As expected, significant over time changes in BW were observed in male and female mice, respectively (**Figures 2A, B**) (Male F_12, 108 _= 86.14, *p* < 0.01; Female F_12, 72 _= 69.48, *p* < 0.01; repeated measures two-way (sampling day × treatment) ANOVA). This is because swapping high preference HFD to low preference standard chow decreased overall energy intake.

KKT administered male mice showed larger decline of BW (**Figure 2A**) (F_1, 108 _= 100.56, *p* < 0.01) and food intake (**Figure 2C**) (F_1, 99 _= 38.13, *p* < 0.01) than those of control on day4 and day5 in the six days period of standard diet administration. However, decreased food intake was not observed for female mice (**Figure 2D**); therefore, the BW decrease caused by KKT administration under a standard diet was observed only in male mice (**Figure 2A**).

Next, in order to identify the effect of KKT on sucrose preference, control (water) and 30% sucrose solution were offered for another six days after standard chow administration. Although there were no differences in water intake between the control and KKT groups in both male and female mice (**Figures 2E, F**) (Male F_1, 45 _= 0.29, *p* > 0.05; Female F_1, 30 _= 5.27, *p* > 0.05), KKT administration induced a decrease in sucrose intake in female mice only (**Figures 2G, H**) (Male F_1, 45 _= 1.08, *p* > 0.05; Female F_1, 30 _= 24.57, *p* < 0.01). During this sucrose offered period, a decline of food intake (**Figure 2D**) (F_1, 66 _= 11.31, *p* < 0.01) and BW (**Figure 2B**) (F_1, 72 _= 25.78, *p* < 0.01) was observed in both male and female mice (**Figures 2A, B**). Analyses of percentage of sucrose intake ([sucrose intake]/[water + sucrose intake]), showed no changes in male mice (**Figure 2I**) (F_1, 45 _= 0.66, *p* > 0.05), while KKT administration significantly decreased this ratio in female mice (**Figure 2J**) (F_1, 30 _= 13.67, *p* < 0.01).

### 3.2 KKT treatment decreases inflammation induced by HFD feeding

Previous results showed that oral KKT administration decreased food intake and BW during first 6 days in only male mice. The result indicates that the impacts of KKT on regulation of feeding and BW are larger in male mice on a standard chow diet. Therefore, we examined systemic inflammation and inflammatory cytokines expressions in liver and adipose tissue of male mice.

Consistent with previous reports, the expressions of IL-6 and OXTR mRNA were significantly increased, and TNFα tended to increase in mesenteric adipose tissues of mice after being fed a HFD (**Supplementary Figures 1A, B**).

**Figure 3:**
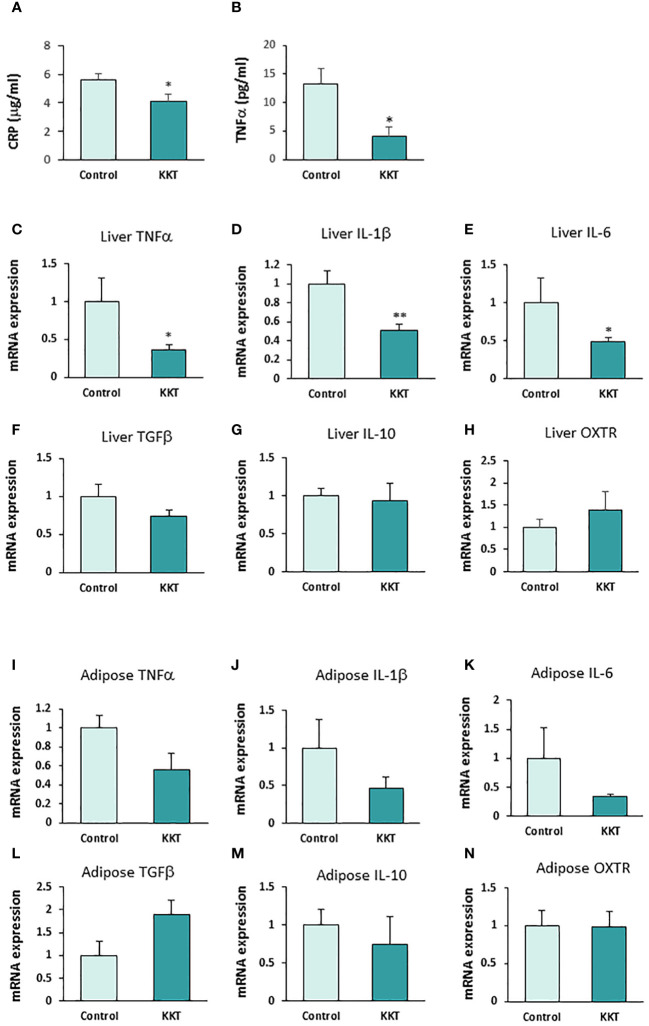
KKT suppresses pro-inflammatory cytokines. **(A, B)** Plasma high-sensitive C-reactive protein (hs-CRP) **(A)** and TNFα **(B)** concentration after KKT oral administration for 12 days in mice with HFD-induced obesity. * P < 0.05, unpaired t-test. *n* = 5, 6. **(C–H)** mRNA expression of pro-inflammatory cytokines **(C-E)**, anti-inflammatory cytokines **(F, G)** and oxytocin receptors (OXTR) **(H)** in liver after KKT oral administration for 12 days in HFD induced obese mice. *P < 0.05, **P < 0.01, unpaired t-test. *n* = 4–6. **(I–N)** mRNA expression of pro-inflammatory cytokines **(I-K)**, anti-inflammatory cytokines **(L, M)** and oxytocin receptors (OXTR) **(N)** in adipose tissues from mesenteric fat after KKT oral administration for 12 days in HFD induced obese mice. *P < 0.05, **P < 0.01, unpaired t-test. *n* = 5–6.

Twelve days of oral administration of 500 mg/kg KKT in the HFD-fed mice significantly decreased plasma hs-CRP (**Figure 3A**; T = 2.26, df = 9, *p* < 0.05) and TNFα (**Figure 3B**; T = 2.83, df = 9, *p* < 0.05), compared with the control group. The hs-CRP were 5.58 ± 0.42 μg/ml in the control group and 4.06 ± 0.50 μg/ml in the KKT administered group. TNFα was 13.20 ± 2.86 pg/ml in the control group and 3.98 ± 1.80 pg/ml in the KKT administered group.

In addition, oral administration of KKT in the HFD-fed mice significantly decreased the expression of mRNA of pro-inflammatory cytokines, TNFα (1.0 ± 0.3 in control, 0.36 ± 0.07 in KKT) **Figure 3C**; T = 2.76, df = 9, *p* < 0.05), IL-1β (1.0 ± 0.14 in control, 0.5 ± 0.06 in KKT) (**Figure 3D**; T = 3.50, df = 9, *p* < 0.01) and IL-6 (1.0 ± 0.3 in control, 0.49 ± 0.05 in KKT) (**Figure 3E**; T = 2.80, df = 9, *p* < 0.05) mRNA expression in the liver. On the other hand, there were no significant differences in anti-inflammatory cytokines, TGFβ (1.0 ± 0.16 in control, 0.73 ± 0.08 in KKT) (**Figure 3F**; T = 1.66, df = 9, *p* > 0.05), IL-10 (1.0 ± 0.61 in control, 0.94 ± 0.23 in KKT) (**Figure 3G**; T = 0.2, df = 8, *p* > 0.05) and OXTR (1.0 ± 0.18 in control, 1.40 ± 0.41 in KKT) (**Figure 3H**; T = 1.06, df = 9, *p* > 0.05) mRNA expression between the control and KKT group liver tissue samples.

In the mesenteric adipose tissue, although mRNA of pro-inflammatory cytokines in the KKT group tended to decrease, there were no significant differences between the control and KKT groups (TNFα, 1.0 ± 0.37 in control, 0.56 ± 0.17 in KKT [**Figure 3I**; T = 1.20, df = 9, *p* = 0.29], IL-1β, 1.0 ± 0.13 in control, 0.46 ± 0.15 in KKT [**Figure 3J**; T = 1.10, df = 9, *p* = 0.30] and IL-6, 1.0 ± 0.52 in control, 0.33 ± 0.04 in KKT [**Figure 3K**; T = 1.67, df = 9, *p* = 0.13]). Similar with the liver tissue samples, there were no significant differences in anti-inflammatory cytokines and OXTR mRNA between the control and KKT groups (TGFβ, 1.0 ± 0.3 in control, 1.90 ± 0.32 in KKT [**Figure 3L**; T = 1.61, df = 9, *p* > 0.05], IL-10, 1.0 ± 0.30 in control, 0.71 ± 0.41 in KKT [**Figure 3M**; T = 0.5, df = 9, *p* > 0.05] and OXTR, 1.0 ± 0.17 in control, 0.98 ± 0.06 in KKT [**Figure 3N**; T = 0.10, df = 8, *p* > 0.05]).

These results indicate that oral administration KKT decreases both systemic inflammation, as is reflected by decreased CRP and TNFα plasma concentrations, and decreased expression of the pro-inflammatory cytokines TNFα, IL-1β and IL-6 in liver.

### 3.3 KKT administration decreases sucrose intake in lean *Oxt* ^-/-^ mice

The results in **Figure 2** showed that oral KKT administration decreased sucrose intake only in female mice. This result indicates that the impacts of KKT on regulation of sucrose intake are more robust in female mice, and thus further studies were performed on female mice alone. Previous work from our laboratory showed that KKT exerts its effect through the activation of OXTRs (26). Thus, in order to directly examine the relationship between OXTR signaling, KKT and sucrose preference, a similar experiment (**Figure 1B**) was performed in lean WT and *Oxt* ^-/-^ middle-aged female mice (35-42wks) on a SD. The initial BWs were 26.33 ± 1.2 g and 24.7 ± 0.34 g, in the WT and *Oxt* ^-/-^mice, respectively, with no significant differences (T = 1.29, df = 8, *p* > 0.05).

**Figure 4:**
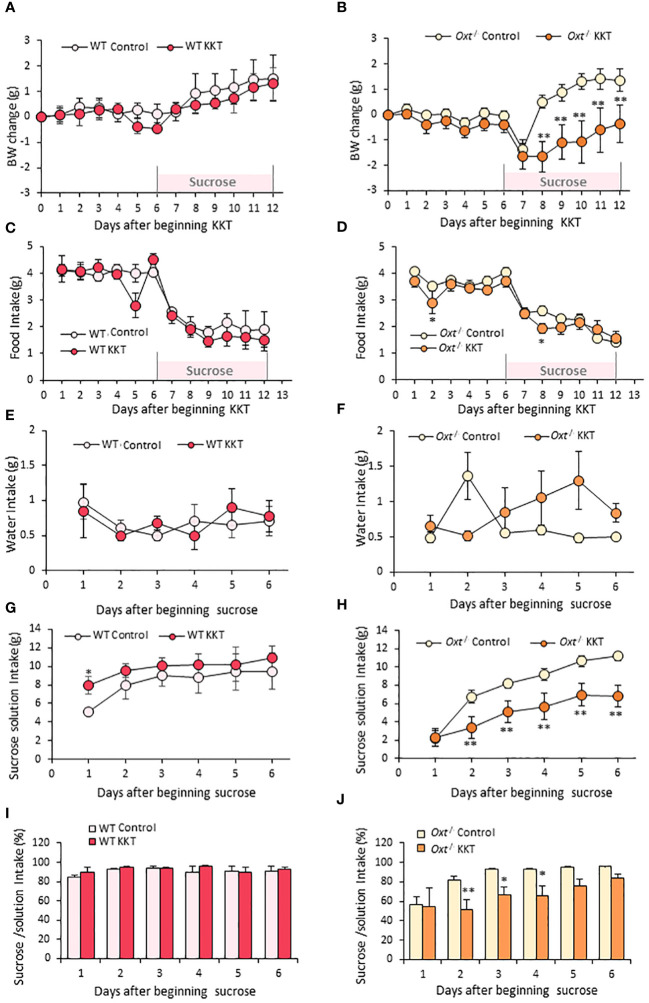
KKT suppresses sucrose intake in *Oxt* ^-/-^ lean female mice. **(A–D)** Body weight change **(A, B)** and food intake **(C, D)** during oral KKT administration in lean WT **(A, C)** and lean *Oxt* ^-/-^ **(B, D)** mice. * P < 0.05, **P < 0.01, Repeated measures two-way ANOVA two-way ANOVA followed by Tukey’s multiple range test. **(E–H**) Water intake **(E, F)** and sucrose solution intake **(G, H)** during sucrose providing term in lean WT **(E, G)** and lean *Oxt* ^-/-^ female **(F, H)** mice. * P < 0.05, **P < 0.01, Repeated measures two-way ANOVA two-way ANOVA followed by Tukey’s multiple range test. **(I, J)** The ratio of sucrose intake ([sucrose intake]/[water + sucrose intake] × 100) in lean WT **(I)** and lean *Oxt* ^-/-^ female **(J)** mice. * P < 0.05, **P < 0.01, Repeated measures two-way ANOVA followed by Tukey’s multiple range test. **(A, C, E, G, I)** *n* = 4, 4. **(B, D, F, H, J)** *n* = 10, 10.

Similar to the obese female mice, KKT administration did not affect BW in the lean WT and *Oxt* ^-/-^ female mice in the first six days (**Figures 4A, B**). However, KKT slightly but significantly decreased food intake in the *Oxt* ^-/-^ mice on day 2 and 8 of KKT administration (**Figure 4D**) (F _1,198 _= 6.08, *p* < 0.05), but showed no significant differences in WT (**Figure 4C**) (F_1, 66_ = 2.37, *p* > 0.05). There were no differences in water intake in the lean WT and *Oxt* ^-/-^lean mice with or without KKT treatment (**Figures 4E, F**) (WT F_1, 30 = _0.01, *p* > 0.05; *Oxt* ^-/-^ F_1, 90 _= 2.01, *p* > 0.05). However, sucrose intake was decreased in KKT administered lean *Oxt* ^-/-^ mice (**Figure 4H**) (F_1, 90 _= 73.29, *p* < 0.01) while a slight increase was observed on day 1 of KKT administration in WT mice (**Figure 4G**) (F_1,30 _= 9.99, *p* < 0.01). Together with declined food and sucrose intake, BW was also decreased in the KKT-administered *Oxt* ^-/-^mice (**Figure 4B**) (F_1, 216 _= 115.27, *p* < 0.01). Although there were no differences in % of sucrose intake between the control and KKT groups in the WT mice (**Figure 4I**) (F_1. 30 = _2.06, *p* > 0.05), KKT significantly decreased % of sucrose intake in the *Oxt* ^-/-^ mice (**Figure 4J**) (F_1, 90 = _19.84, *p* < 0.01). This result would support the idea that, in the absence of native oxytocin, KKT acts as an agonist of OXTR to exerts its sucrose preference suppressing effect in female.

The expression of OXTR in the hypothalamus, hippocampus and cerebral cortex were examined in the WT and *Oxt* ^-/-^ female mice by qRT-PCR. OXTR mRNA expression in the cerebral cortex was significantly increased (T = 5.19, df = 4, *p* < 0.01) (**Supplementary Figure 2C**) and tended to increase in the hippocampus (T = 2.27, df = 4, *p* = 0.08) (**Supplementary Figure 2B**). There were no significant differences in the hypothalamus (T = 0.85, df = 4, *p* = 0.44) (**Supplementary Figure 2A**). These results would further suggest that the mechanism underpinning the observed decline of preference for sucrose induced by KKT administration in *Oxt* ^-/-^mice is OXTR dependent, and mediated via the limbic system.

## 4 Discussion

The present study demonstrated that KKT ameliorates inflammation induced by obesity, decreases food intake in obese middle-aged males, and sucrose intake in obese middle-aged females. The decline of food intake in males and sucrose intake in females most likely contributes to the reduction of BW observed for both sexes.

Obesity is strongly associated with metabolic syndrome, which includes abdominal obesity, insulin resistance, hypertension, and dyslipidemia (37). Obesity increases the prevalence of metabolic syndrome and raises the risk of cardiovascular diseases and type 2 diabetes (37). Obesity is typically characterized as the simple accumulation of adipose tissue but recent studies highlighted the contribution of chronic inflammation in adipocytes to pathology (10). It is now accepted that accumulated adipose tissue as the primary source of pro-inflammatory cytokines that induce systemic chronic inflammation (10). This obesity-induced systemic chronic inflammation leads to insulin resistance, diabetes and cardiovascular disease (4). This systemic inflammation is reflected in an increase of CRP that is synthesized and secreted in the liver, under the transcriptional control of IL-6 (6). In obese patients, it is not only CRP secretion, but also dietary factors such as the consumption of high calorie foods which contributes to non-alcoholic fatty liver with inflammation, cirrhosis, and ultimately hepatocellular carcinoma (38). Aging itself also leads to the induction of chronic systemic inflammation. The combination of obesity and aging can lead to further increase of inflammation (9) and the prevalence of obesity is typically higher among middle-aged adults (40-59 yrs) than among younger (20-39 yrs) groups (3).

In the present study, we demonstrate that KKT treatment decreased the plasma inflammation markers hs-CRP and TNFα as well as the expression of pro-inflammatory cytokines in the liver in middle-aged mice. The data suggests that KKT may reduce the production of CRP from the liver and suppress the systemic inflammation and development of fatty liver disease.

To the best of our knowledge, this paper is the first to report the anti-inflammatory effect of KKT. How KKT suppresses inflammation in liver and adipose tissue remains unclear. However, considering our previous report, which demonstrated the capability of KKT to activate OXTR (30), at least two mechanisms can be considered for its anti-inflammation mechanism; first, KKT directly affects hepatocytes and adipocytes; second, KKT affects macrophages within the liver and adipose tissues.

As for the first possible mechanism, it is based on previous reports showing that OXTR is expressed in both hepatocytes and adipocytes (26, 39). OXTR in hepatocytes contribute to the rejuvenation of the liver through activation of autophagy and regeneration of hepatocyte (26) while OXTR in adipocytes regulates lipolysis, and its activation reduces the size of adipocytes (39). Further supporting this work, our previous study also confirmed that chronic oxytocin treatment improves fatty liver and reduces the size of adipocytes (19). Considering these reports, KKT may suppress inflammation by rescuing damaged liver and adipose tissue.

As for the second possibility, KKT may have direct effects on macrophages. OXTR is reported to be expressed on macrophages (40) and is OXTR expression is increased following acute inflammation induced by the lipopolysaccharides (LPS). This increase of OXTR expression in macrophages is known to attenuate inflammatory responses (40).

Considering the decline of IL-6 mRNA expression in liver and plasma hs-CRP induced by KKT administration, the two mechanisms discussed above are both plausible. Our previous study (30) showed that KKT administration increased cytosolic Ca^2+^ in OXTR transfected HEK cells which were abolished in the presence of an OXTR antagonist. Furthermore, we identified 7 chemical components (rutin, ursolic acid, z-butylidenephtalide, sensyunolide-A, P-cymene, [6]-shogaol, [8]-shogaol) from 3 crude drugs (Zizyphi Fructus, Angelicae Acutilobae Radix, Zingiberis Rhizoma) in KKT which act as OXTR agonists. Therefore, these crude drugs may activate OXTR synergistically. However, since KKT is composed of 14 different crude drugs (**Supplementary Table 1**), different mechanisms independent from OXTR could be involved in attenuating inflammation in the liver and adipose tissue. Further studies, including the use of OXTR knockout mice, are therefore needed to better understand the underlying mechanisms of KKT effect.

As for the food and sucrose intake, there were sex differences in the effect of KKT administration. Male mice showed a decrease in food intake, but not in sucrose intake, while female mice showed a decrease in sucrose intake, but not in food intake. There are two mechanisms for food intake regulation. The first is the basal food intake, which plays a crucial role in matching caloric intake with energy expenditure, and is regulated in the medulla oblongata and the hypothalamus (41). The second mechanism is high palatable feeding, for example sucrose, which is regulated in the reward related brain region including the amygdala (42) and ventral tegmental area (43). Our data therefore would suggest that KKT mainly affects basal feeding regulation in male mice, and reward feeding regulation in female mice.

Because oxytocin preferentially suppresses intake of sweet-tasting carbohydrates in rats, mice, monkeys and humans (20, 23, 44–46), our present result suggests that KKT affect sucrose consumption via oxytocin signaling mediated mechanisms. Therefore, we examined the effect of KKT on food intake and sucrose intake by using lean *Oxt* ^-/-^ middle-aged female mice. Interestingly, KKT showed almost no effect on food intake and sucrose intake in lean WT female mice while it suppressed only sucrose intake in lean *Oxt* ^-/-^ female mice. Since KKT is an established OXTR agonist (30), it is therefore plausible that KKT suppressed sucrose intake via oxytocin induced signaling cascades. Interestingly, sucrose intake was significantly decreased for the first two days of access to sucrose in untreated lean *Oxt* ^-/-^mice (**Figure 3H**) when compared to untreated lean WT littermates (**Figure 3G**), and untreated lean *Oxt* ^-/-^mice only caught up with WT littermates on the third day of access to sucrose. This would imply that either taste or reward systems are impacted by the loss of oxytocin in these mice, and untreated lean *Oxt* ^-/-^mice only developed a taste for sucrose over time. This sweet palatable food intake regulation by oxytocin is most likely mediated by OXTR expressed in reward-related brain regions (23). A previous human study showed that intranasally administered oxytocin reduced post-stress sweet snack intake in female participants (46). This mechanism may be explained through the actions of OXTR in dopamine neurons in reward related brain regions, such as the ventral tegmental area, nucleus accumbens by attenuating its excitatory input (23, 47).

It is possible that the sex differences for the effect of KKT may be caused by the different expression levels of OXTR in the brain (48). OXTR expression is reported to be higher in the lateral septum area in male than in female mice, but higher in the amygdala in female than male mice (48). Further studies about sex differences in OXTR expression and the effect of KKT are needed.

One of the main questions raised by the current study is that KKT administration is shown to be effective only in obese mice and its effect observed in lean *Oxt* ^-/-^ mice is surprising. This effect may be explained by the observed increase of OXTR expression levels in the cerebral cortex and hippocampus as a compensatory mechanism in response to the loss of functioning oxytocin. It is well established that obesity increases OXTR mRNA expression level in adipose tissue and the brain (49, 50), an observation further supports by our own finding that HFD feeding increases OXTR mRNA expression in adipose tissue (**Supplementary Figure 1D**). Similarly, OXTR mRNA was significantly increased in cerebral cortex, and tended to increase in hippocampus (*p* = 0.085) in middle-aged lean *Oxt^-/-^* mice (**Supplementary Figures 2B, C**). This is in line with the previous article, which reported the deficient of oxytocin lead to OXTR upregulation and/or increased sensitivity (51). Earlier studies reported decreased plasma oxytocin levels in obese, type2 diabetic, and metabolic syndrome patients and aged mice (24, 52, 53). Although we did not measure plasma oxytocin levels in our middle-aged obese model, the decline of plasma oxytocin levels is speculated. Because our middle-aged obese model contains the critical factors, “aging” and “obesity”, that decline of plasma oxytocin levels. Therefore, this phenomenon may be a compensatory mechanism to reflect the decline of oxytocin levels as a result of obesity, aging or complete loss of oxytocin in *Oxt* ^-/-^ mice. Previous reports and work from our laboratory showed that anti-obesity and the anorexigenic effect of oxytocin depended on the severity of obesity. Oxytocin effects on food intake and body weight are directly proportional to mice body weight (54). These results are in agreement with what we have shown here. Also, OXTR mRNA and protein expression are reported to be dramatically upregulated with LPS induced inflammation in macrophages (40). Since KKT moderately activates OXTR (30), the effect of KKT may be amplified when OXTR expression is upregulated in conditions such as inflammation, obesity, aging and under the condition that plasma oxytocin becoming low.

To date, there are no reports showing the effect of KKT on chronic inflammation. However, a recent clinical report showed that KKT attenuated LPS (Lipopolysaccharide) induced depressive behavior, such as loss of object exploration, social interaction deficit, and depressive-like behavior (55). However, it was considered that this effect of KKT was caused via attenuation of neural activity and not by attenuating inflammation (55). However, this paper did not investigate the effect of chronic inflammation. Recent studies highlighted that many diseases, including depression, cardiovascular disease, and cancer are strongly associated with the state of chronic inflammation (38, 56, 57).

An interesting extension of this study would be to test the effect of KKT on younger mice. The current study specifically investigates the effect of KKT administration on middle-aged mice, to reflect the age of the patients typically prescribed this herbal supplement. It would be of interest to see if these effects can also be seen in younger obese mice, which has a different metabolic profile compared to older mice. Since the combination of obesity and aging additively leads to systemic inflammation (9), it can be speculated that in younger obese mice, there could be less inflammation and less decline in plasma oxytocin levels. In this study, it was therefore important to consider the age of mice as a contributing factor as the effects of KKT on BW change and food intake, inflammation and sucrose preferences may be different in younger mice compared to middle-aged mice.

Furthermore, a decline of BW gain in both male and female mice in the KKT treated group was observed. The possible explanation of decreasing BW gain can be considered to be due to the increase in energy expenditure and thermogenesis, as well as decline of food or sucrose intake. Systemic oxytocin treatment increased energy expenditure (19) and oxytocin KO mice show decreased energy expenditure and thermogenesis (16, 58). Thus, the effects of KKT on thermogenesis should be considered as a possible contributing factor in this study.

In summary, the results of the present study show the inhibitory effect of KKT administration on both appetite and chronic inflammation in middle-aged obese mice. Since KKT can regulate inflammatory cytokines secretion, KKT may have a clinical advantage in the treatment of dietary and aging-related chronic inflammation disorders including obesity, steatohepatitis, as well as a treatment of neuropsychological stress disorders.

## Data availability statement

The original contributions presented in the study are included in the article/**Supplementary Material**. Further inquiries can be directed to the corresponding authors.

## Ethics statement

The animal study was approved by Ethical Committee of Fukushima Medical University for Genetic Experiments and Animal Care and Use. The study was conducted in accordance with the local legislation and institutional requirements.

## Author contributions

YM: Writing – review & editing, Writing – original draft, Validation, Supervision, Project administration, Methodology, Investigation, Funding acquisition, Formal analysis. SY: Writing – review & editing, Methodology, Investigation, Formal analysis. MY: Writing – review & editing, Methodology, Investigation. SM: Writing – review & editing, Supervision. TO: Writing – review & editing, Supervision, Investigation. HO: Writing – review & editing. KM: Writing – review & editing. SH: Writing – review & editing, Resources. KN: Writing – review & editing, Resources. MA: Writing – review & editing, Supervision, Formal analysis. Hd: Writing – review & editing, Writing – original draft, Supervision. KS: Writing – review & editing, Writing – original draft, Supervision, Funding acquisition.
