# Supplementary material for: Traditional Japanese medicine Kamikihito ameliorates sucrose preference, chronic inflammation and obesity induced by a high fat diet in middle-aged mice
Source: Front Endocrinol (Lausanne). 2024 Apr 29;15:1387964. doi: 10.3389/fendo.2024.1387964 (PMC11089234; doi:10.3389/fendo.2024.1387964)

*Supplemental information*

**Traditional Japanese medicine Kamikihito ameliorates sucrose preference, chronic inflammation and obesity induced by a high fat diet in middle-aged mice**

Yuko Maejima, Shoko Yokota, Megumi Yamachi, Shingen Misaka, Tomoyuki Ono, Hiroaki Oizumi, Keita Mizuno, Tomoyuki Ono, Shizu Hidema, Katsuhiko Nishimori,　Masato Aoyama, Heidi de Wet, Kenju Shimomura

^
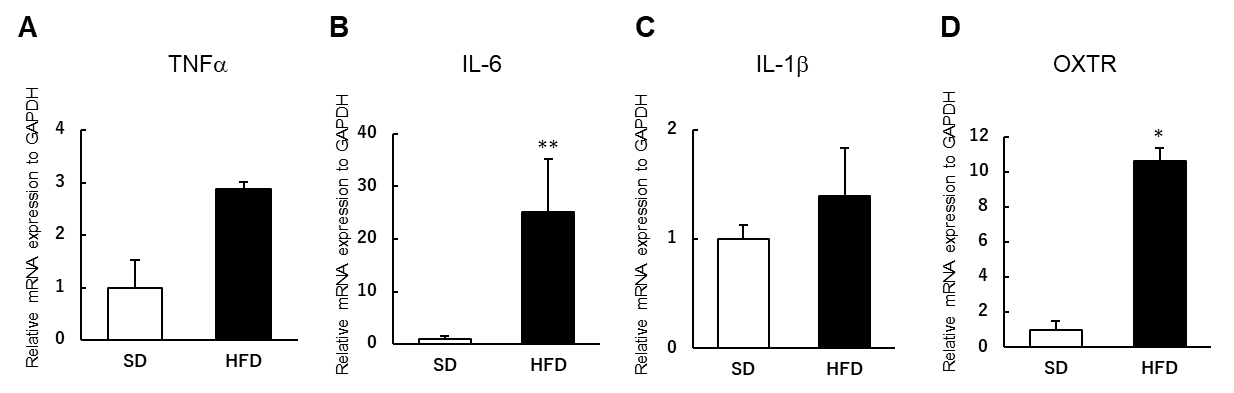
^

Supplementary Figure 1

A–D: mRNA expression of proinflammatory cytokines (A–C) and oxytocin receptors (OXTR) (D) in mesenteric fat after high fat diet feeding for 24 weeks. * P < 0.05, ** P < 0.01, unpaired t-test. n = 4–5.

Six-week-old C57BL/6J male mice were fed standard (SD) chow or a high fat diet (HFD) for 24 weeks, and mRNA expression in mesenteric fat was measured. The methods and primers were the same as those described in the Materials and Methods section (2.3. Quantitative real‑time PCR).

^
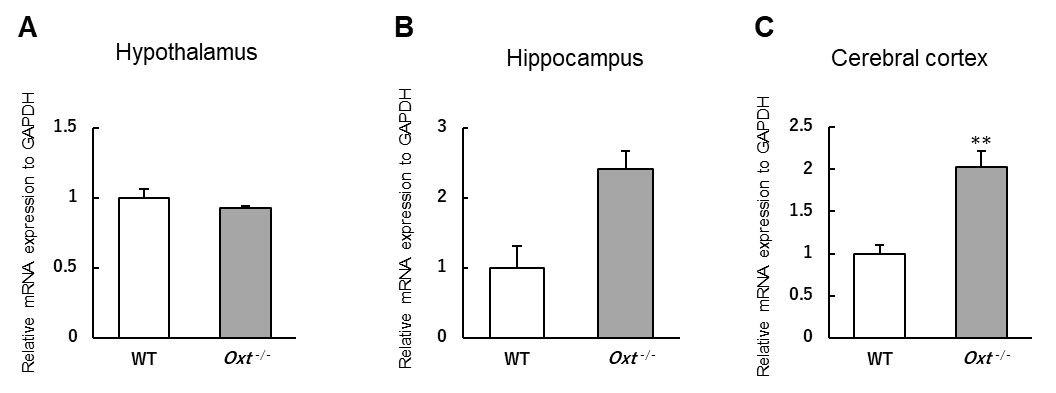
^

Supplementary Figure 2

A–C: mRNA expression of oxytocin receptors (OXTR) in the hypothalamus (A), hippocampus (B) and cerebral cortex (C) in wild type (WT) and oxytocin deficient (*Oxt*^-/-^) mice.

WT and *Oxt*^-/-^ female mice (50 weeks) were anesthetized and their brain was removed. The brain sections (0.14 mm to -2.06 mm from bregma) were made, and the hippocampus, hypothalamus and cerebral cortex from these sections were dissected under the microscope. The methods and primers were the same as those described in the Materials and Methods section (2.3. Quantitative real‑time PCR). ** P < 0.01, unpaired t-test. n = 3, 3.


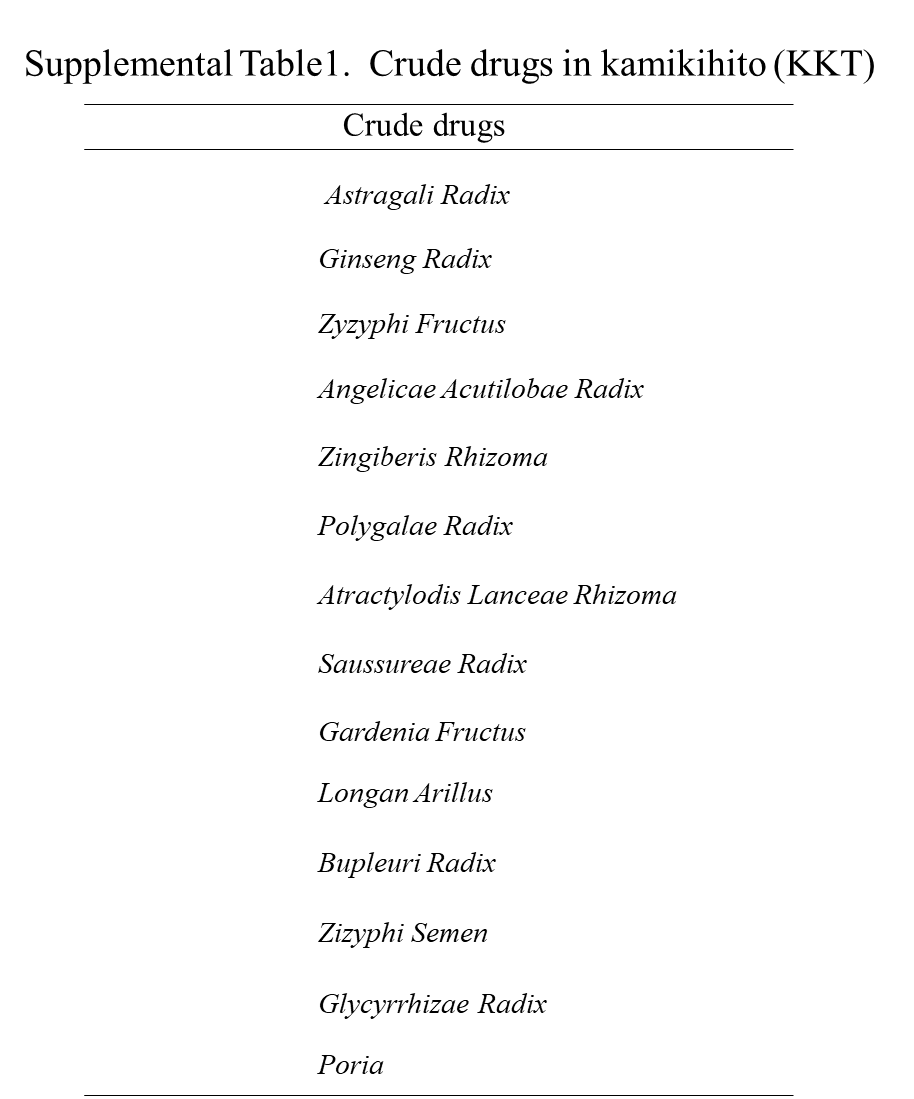

Supplement: Supplementary file 1 [file DataSheet_1.docx]
